# Supplementary material for: Building Multivariate Molecular Imaging Brain Atlases Using the NeuroMark PET Independent Component Analysis Framework
Source: bioRxiv. 2025 Feb 23:2025.02.18.638362. Preprint. [Version 1] doi: 10.1101/2025.02.18.638362 (PMC11870563; doi:10.1101/2025.02.18.638362)
Supplement: Supplement 2 [file media-2.pdf]

Title: Building Multivariate Molecular Imaging Brain Atlases Using the NeuroMark PET Independent Component Analysis Framework

Authors: Cyrus Eierud[a], Martin Norgaard[b,c], Murat Bilgel[d], Helen Petropoulos[a], Zening Fu[a], Armin Iraji[a], Granville J. Matheson[e,f,g], Melanie Ganz[c,h], Cyril Pernet[h], Vince D. Calhoun[a,i,j] for the Alzheimer's Disease Neuroimaging Initiative*

Supplementary Material

Table S1. Descriptive statistics for the SUVR values in the DKT ROIs: mean (μ), standard deviation (σ), association with age using beta (β) and the FDR corrected p (q) for FBB and FBP. In the q columns some DKT ROIs are excluded: because they are not gray matter (x-non-gm), because they were used for SUVR reference or because the age association was negative (x-neg). Acronyms: left (L.); right (R.).

DKT Label	μ FBB	σ FBB	β FBB	q FBB	μ FBP	σ FBP	β FBP	q FBP
L. Cerebral White Matter	2.00	0.17	0.114	x-non-gm	2.02	0.23	-0.118	x-non-gm
L. Cerebellum White Matter	2.02	0.16	0.163	x-non-gm	1.90	0.20	-0.167	x-non-gm
L. Cerebellum Cortex	1.00	0.01	0.067	reference	1.00	0.01	-0.206	reference
L. Thalamus	1.52	0.16	0.127	1.00E-01	1.44	0.16	-0.087	x-neg
L. Caudate	1.18	0.15	0.047	5.50E-01	1.23	0.17	0.056	3.91E-01
L. Putamen	1.48	0.18	0.274	7.08E-04	1.51	0.20	0.130	4.88E-02
L. Pallidum	1.93	0.19	0.206	8.66E-03	1.82	0.21	-0.133	x-neg
Brain Stem	1.72	0.14	0.224	4.47E-03	1.60	0.16	-0.081	x-neg
L. Hippocampus	1.26	0.10	0.001	9.85E-01	1.23	0.12	-0.320	x-neg
L. Amygdala	1.16	0.11	0.190	1.51E-02	1.17	0.14	0.037	5.41E-01
CSF	0.85	0.16	-0.370	x-non-gm	0.82	0.17	-0.441	x-non-gm
L. Accumbens area	1.21	0.27	0.162	3.73E-02	1.25	0.28	0.159	2.12E-02
L. VentralDC	1.82	0.16	0.183	1.88E-02	1.70	0.18	-0.17	x-neg
L. choroid plexus	1.04	0.19	-0.287	x-non-gm	0.97	0.19	-0.387	x-non-gm
R. Cerebral White Matter	2.02	0.18	0.147	x-non-gm	2.03	0.23	-0.111	x-non-gm
R. Cerebellum White Matter	2.05	0.16	0.14	x-non-gm	1.92	0.20	-0.152	x-non-gm
R. Cerebellum Cortex	1.00	0.01	-0.066	reference	1.00	0.01	0.208	reference
R. Thalamus	1.52	0.16	0.15	5.37E-02	1.44	0.16	-0.074	x-neg
R. Caudate	1.20	0.16	0.085	2.74E-01	1.25	0.17	0.043	4.93E-01
R. Putamen	1.48	0.19	0.332	3.21E-04	1.51	0.19	0.137	3.82E-02
R. Pallidum	1.93	0.19	0.239	2.62E-03	1.82	0.21	-0.135	x-neg
R. Hippocampus	1.28	0.09	0.02	8.04E-01	1.25	0.12	-0.344	x-neg
R. Amygdala	1.19	0.12	0.223	4.70E-03	1.18	0.14	0.016	7.80E-01
R. Accumbens area	1.23	0.27	0.235	2.93E-03	1.27	0.27	0.145	3.02E-02
R. VentralDC	1.79	0.16	0.15	5.34E-02	1.68	0.18	-0.187	x-neg
R. choroid plexus	1.07	0.17	-0.272	x-non-gm	1.02	0.17	-0.365	x-non-gm
AirCavity	0.50	0.10	0.123	x-non-gm	0.53	0.12	0.069	x-non-gm
Skull	0.82	0.18	-0.07	x-non-gm	0.74	0.18	-0.092	x-non-gm
Vermis	0.95	0.04	0.107	1.69E-01	0.95	0.05	-0.108	x-neg

Pons	2.17	0.19	0.256	1.45E-03	2.04	0.24	-0.109	x-neg
CSF Extra Cerebral	0.89	0.15	0.102	x-non-gm	0.88	0.15	0.059	x-non-gm
Head Extra Cerebral	1.02	0.15	-0.106	x-non-gm	1.04	0.18	-0.118	x-non-gm
L. Banks of Sup. Temp. Sulcus	1.47	0.28	0.258	1.36E-03	1.57	0.32	0.137	3.82E-02
L. Caudal Anterior Cingulate	1.39	0.25	0.298	4.30E-04	1.44	0.26	0.147	2.90E-02
L. Caudal Middle Frontal	1.34	0.24	0.259	1.31E-03	1.40	0.28	0.2	8.22E-03
L. Cuneus	1.29	0.16	0.205	8.81E-03	1.33	0.21	0.046	4.72E-01
L. Entorhinal Cortex	1.10	0.13	0.211	7.13E-03	1.07	0.13	0.058	3.86E-01
L. Fusiform Gyrus	1.26	0.20	0.251	1.65E-03	1.31	0.24	0.128	5.03E-02
L. Inferior Parietal Lobule	1.34	0.26	0.267	9.41E-04	1.40	0.29	0.131	4.78E-02
L. Inferior Temporal Gyrus	1.28	0.24	0.280	6.18E-04	1.33	0.28	0.168	2.01E-02
L. Isthmus of Cingulate Gyrus	1.40	0.25	0.322	3.21E-04	1.42	0.26	0.143	3.23E-02
L. Lateral Occipital Cortex	1.31	0.17	0.282	6.18E-04	1.36	0.25	0.081	2.17E-01
L. Lateral Orbitofrontal Cortex	1.33	0.24	0.274	7.14E-04	1.37	0.26	0.184	1.25E-02
L. Lingual Gyrus	1.21	0.14	0.203	9.38E-03	1.25	0.21	0.052	4.27E-01
L. Medial Orbitofrontal Cortex	1.27	0.27	0.251	1.65E-03	1.31	0.28	0.193	8.22E-03
L. Middle Temporal Gyrus	1.25	0.24	0.274	7.08E-04	1.29	0.27	0.152	2.55E-02
L. Parahippocampal Gyrus	1.15	0.17	0.237	2.73E-03	1.15	0.18	0.051	4.28E-01
L. Paracentral Lobule	1.34	0.20	0.166	3.41E-02	1.37	0.23	0.077	2.29E-01
L. Pars Opercularis	1.29	0.24	0.255	1.47E-03	1.35	0.26	0.157	2.22E-02
L. Pars Orbitalis	1.28	0.25	0.253	1.56E-03	1.29	0.27	0.169	2.01E-02
L. Pars Triangularis	1.34	0.24	0.248	1.83E-03	1.38	0.27	0.147	2.90E-02
L. Pericalcarine Cortex	1.38	0.18	0.181	2.04E-02	1.48	0.26	0.108	9.79E-02
L. Postcentral Gyrus	1.24	0.18	0.139	7.37E-02	1.27	0.20	0.057	3.91E-01
L. Posterior Cingulate	1.41	0.28	0.288	5.68E-04	1.46	0.30	0.153	2.55E-02
L. Precentral Gyrus	1.31	0.16	0.197	1.19E-02	1.34	0.19	0.090	1.71E-01
L. Precuneus	1.37	0.31	0.265	1.01E-03	1.44	0.33	0.138	3.82E-02
L. Rostral Anterior Cingulate	1.30	0.28	0.280	6.18E-04	1.36	0.30	0.166	2.01E-02
L. Rostral Middle Frontal	1.32	0.29	0.243	2.22E-03	1.37	0.31	0.175	1.63E-02
L. Superior Frontal Gyrus	1.27	0.26	0.250	1.70E-03	1.32	0.27	0.198	8.22E-03
L. Superior Parietal Lobule	1.29	0.23	0.194	1.30E-02	1.35	0.26	0.107	9.93E-02
L. Superior Temporal Gyrus	1.24	0.21	0.238	2.62E-03	1.28	0.24	0.129	5.01E-02
L. Supramarginal Gyrus	1.30	0.25	0.224	4.47E-03	1.36	0.27	0.116	7.42E-02
L. Frontal Pole	1.13	0.30	0.253	1.56E-03	1.13	0.30	0.193	8.22E-03
L. Temporal Pole	1.14	0.16	0.230	3.49E-03	1.14	0.18	0.161	2.06E-02
L. Transverse Temporal Gyrus	1.28	0.21	0.153	5.03E-02	1.34	0.24	0.100	1.23E-01
L. Insula	1.24	0.20	0.235	2.95E-03	1.27	0.21	0.122	5.97E-02
R. Banks of Sup. Temp. Sulcus	1.48	0.28	0.298	4.30E-04	1.58	0.32	0.147	2.90E-02
R. Caudal Anterior Cingulate	1.36	0.26	0.275	7.08E-04	1.41	0.27	0.122	5.99E-02
R. Caudal Middle Frontal	1.34	0.25	0.275	7.08E-04	1.40	0.27	0.195	8.22E-03
R. Cuneus	1.28	0.16	0.242	2.30E-03	1.31	0.20	0.042	4.93E-01
R. Entorhinal Cortex	1.10	0.13	0.313	3.21E-04	1.07	0.13	0.030	6.19E-01
R. Fusiform Gyrus	1.26	0.22	0.308	3.21E-04	1.29	0.23	0.123	5.97E-02
R. Inferior Parietal Lobule	1.34	0.28	0.282	6.18E-04	1.40	0.29	0.141	3.33E-02

R. Inferior Temporal Gyrus	1.27	0.25	0.333	3.21E-04	1.32	0.26	0.175	1.63E-02
R. Isthmus of Cingulate Gyrus	1.40	0.26	0.310	3.21E-04	1.42	0.26	0.144	3.19E-02
R. Lateral Occipital Cortex	1.33	0.20	0.292	5.03E-04	1.35	0.24	0.100	1.23E-01
R. Lateral Orbitofrontal Cortex	1.34	0.25	0.311	3.21E-04	1.38	0.25	0.179	1.52E-02
R. Lingual Gyrus	1.22	0.17	0.215	6.32E-03	1.23	0.18	0.051	4.28E-01
R. Medial Orbitofrontal Cortex	1.28	0.29	0.295	4.70E-04	1.33	0.29	0.193	8.22E-03
R. Middle Temporal Gyrus	1.26	0.25	0.309	3.21E-04	1.31	0.26	0.161	2.06E-02
R. Parahippocampal Gyrus	1.17	0.18	0.310	3.21E-04	1.17	0.18	0.050	4.28E-01
R. Paracentral Lobule	1.34	0.20	0.193	1.36E-02	1.38	0.23	0.086	1.91E-01
R. Pars Opercularis	1.31	0.25	0.280	6.18E-04	1.36	0.26	0.164	2.01E-02
R. Pars Orbitalis	1.29	0.26	0.302	4.27E-04	1.30	0.27	0.165	2.01E-02
R. Pars Triangularis	1.35	0.26	0.273	7.14E-04	1.38	0.27	0.151	2.55E-02
R. Pericalcarine Cortex	1.37	0.20	0.215	6.32E-03	1.45	0.25	0.080	2.18E-01
R. Postcentral Gyrus	1.24	0.18	0.164	3.55E-02	1.27	0.20	0.041	4.97E-01
R. Posterior Cingulate	1.40	0.29	0.313	3.21E-04	1.46	0.29	0.153	2.55E-02
R. Precentral Gyrus	1.31	0.17	0.204	9.36E-03	1.35	0.19	0.080	2.18E-01
R. Precuneus	1.37	0.30	0.272	7.37E-04	1.43	0.32	0.126	5.33E-02
R. Rostral Anterior Cingulate	1.31	0.29	0.290	5.21E-04	1.38	0.29	0.159	2.12E-02
R. Rostral Middle Frontal	1.34	0.30	0.286	5.80E-04	1.38	0.31	0.167	2.01E-02
R. Superior Frontal Gyrus	1.28	0.26	0.279	6.37E-04	1.32	0.27	0.195	8.22E-03
R. Superior Parietal Lobule	1.29	0.22	0.240	2.56E-03	1.34	0.26	0.080	2.18E-01
R. Superior Temporal Gyrus	1.24	0.21	0.280	6.18E-04	1.27	0.23	0.128	5.07E-02
R. Supramarginal Gyrus	1.29	0.23	0.259	1.31E-03	1.35	0.26	0.111	8.84E-02
R. Frontal Pole	1.17	0.31	0.287	5.74E-04	1.17	0.30	0.212	8.22E-03
R. Temporal Pole	1.14	0.17	0.300	4.30E-04	1.13	0.17	0.103	1.14E-01
R. Transverse Temporal Gyrus	1.26	0.20	0.220	5.14E-03	1.31	0.22	0.060	3.77E-01
R. Insula	1.25	0.21	0.293	5.01E-04	1.26	0.21	0.117	7.11E-02
